# Digital technologies: students’ expectations and experiences during their transition from high school to university

**DOI:** 10.1007/s10639-022-11184-4

**Published:** 2022-07-07

**Authors:** Therese Keane, Tanya Linden, Paul Hernandez-Martinez, Andreea Molnar, Aaron Blicblau

**Affiliations:** 1 Swinburne University of Technology, PO Box 218, Hawthorn, VIC Australia; 2 Faculty of Engineering and IT, The University of Melbourne, 3010 Parkville, VIC Australia

**Keywords:** Digital technologies, Expectations, Attitudes, Transition to tertiary education

## Abstract

University students’ expectations of digital technologies in their studies are greatly influenced by their previous exposure both within the secondary school classroom and in their private lives. These expectations often play a powerful role in their approaches and learning strategies in their first-year university classes. In this work, we investigated students’ expectations and utilisation of digital technologies in their transition from high school to tertiary studies. A survey encompassing the Expectancy-Disconfirmation Paradigm was conducted amongst second year students across the university. The results showed students expected to use advanced IT technologies and equipment at university. The high expectations were similar regardless of demographic parameters, such as gender, school type or field of studies. The findings also indicated that most students perceived little disconnection between the technology they used in high school and that of university. The majority of students seemed satisfied and even positively surprised by the use of technology in their university courses.

## Introduction

In the 21st century, most learning environments at all levels of education take advantage of technological developments. Different technologies facilitate quality learning and modern-day students can mix and match various offerings, including compulsory ones such as Learning Management Systems (LMS) or library systems, to optional ones that support their individual learning needs. Understanding the effects that individual technologies have on students’ learning is crucial for creating a productive and engaging learning environment (Gosper et al., 2013) and students’ expectations strongly contribute to this understanding. This became even more evident when the world experienced the latest pandemic of COVID 19 and all educational institutions had to move their teaching to online mode with little time to prepare staff and students to this new experience. Even when later in the pandemic the restrictions were relaxed, the return to campus was quite limited and teaching and learning have had to proceed in a blended mode.

In this study we consider digital technologies to include electronic devices, software and any online tools that could be used to enhance students’ learning process and overall learning environment (Drent & Meelissen, 2008; Müller & Mildenberger, 2021). Students’ attitudes towards digital technologies affect how they approach their learning including how they develop their own specific knowledge base and how they set and adjust their unique learning strategies in order to achieve their learning goals.

Upon transitioning from high school to tertiary education, students’ own experiences of digital technologies influence their perceptions and expectations of advanced technologies for the “perfect” tertiary study. What occurs in their introductory first year subjects plays a vital role in their attitudes and response to their learning journey. The impact of their attitudes could be particularly important if there is a significant disconnect between what the students expect to learn and how the course content expects them to engage. This is especially important in the current climate of universities being in different financial positions and their ability to provide technology to support students’ learning. At the university where the data was collected lack of money resulted in freezing all lab computers upgrades. In addition the IT department made some unpopular decisions discontinuing support of some systems used for teaching.

In this climate, this project explores student attitudes and expectations about the use and implementation of digital technologies at tertiary level, as well as how their attitudes and expectations are influenced by their school-based experiences, as summarised in the research question:

*Is there a digital technology disconnect between expectations and actual experiences when students get to first year university from high school?*

Since so little is known about the attitudes and students’ expectations of digital technologies in the tertiary sector, it is important to explore differences in expectations and attitudes of students based on various parameters, namely the type of high school they come from (Government, Independent or Catholic), school location (urban or rural) and university faculty (Health, Arts and Design, or Science, Engineering, and Technology, or Business and Law).

## Literature Review

Over the past 20 years the technology landscape at different levels of education has been changing at a very fast pace. Students’ positive attitudes towards digital technology make it an important tool that could enrich study experience (Henderson et al., 2017; Novikova et al., 2021). There is evidence that students perceive digital technologies as an important component of the learning environment and expect certain level of engagement with technology (Popovici & Mironov, 2015). Therefore educational organisations need to take into consideration students’ expectations of technology when designing learning environments (Hamutoglu et al., 2020). Students make their own decisions on how to use technology at home for their studies and everyday tasks and they bring that experience to their educational environment.

### Technology in schools

Globally, the value of using information technology is highly regarded and schools have made significant efforts to ensure that digital technologies are used in classrooms. Since 2009, the individual access ratio of one computer to one student in many countries has been increasing (OECD, 2020). Students have unprecedented access to digital technologies in and outside of the classroom as technologies become pervasive. In Australia, for example, since 2008, the use of technology in schools has increased due to government policy that directed significant funding to ensure that senior secondary students across all three school sectors (Government, Independent and Catholic) had access to their own portable computers or tablet devices (Keane & Keane, 2020; Rudd et al., 2007). The issue of equity has been largely addressed with the provision of individual access to a mobile device due to their multifunctionality and affordability, however studies have shown that teachers’ pedagogical practices with digital technologies determine how it is used in the classroom (OECD, 2019; Redecker, 2017). Students are encouraged to use digital technologies to access, create and communicate information and ideas, solve problems and work collaboratively. Students are being empowered to develop these skills necessary for future studies and successful careers as well as to give them control over how they learn.

The importance of the Internet cannot be understated. On average 95% of 15-year-old students have access to the internet in OECD countries (OECD, 2019), however some countries have limited Internet infrastructure and as a result their students are strongly disadvantaged. Internet connectivity underpins the accessibility and usability of digital educational resources such as learning management systems, wikis’ and blogs.

### Types of School

Australia’s schooling system is divided into three sectors, each of which has a significant enrolment share - Independent Schools, Catholic Schools and Government Schools. In 2020, there were 4,006,974 students enrolled in schools across Australia, with 65.6% or 2.6 million attending Government schools, 19.4% or 778,605 students attending Catholic schools and 15% or 599,226 students attending Independent schools (Australian Bureau of Statistics, 2021). Unlike Government schools, Catholic schools are part funded by the Government with a co-contribution by parents whereas Independent schools (which incorporate other faiths) depend primarily on private sources for their recurring income (such as fees from parents) along with government funding, whereas throughout much of Europe and New Zealand faith-based schools tend to be part of a state or “integrated” education system, therefore not requiring additional payments for school fees. The type of school can influence the access that students have to technology. Independent or private schools (also known as public schools in Britain) often have access to more resources than Government/State based schools or Catholic Schools, however it should be noted that not all Independent schools serve communities from higher socioeconomic backgrounds (Gonski et al., 2011). On average across OECD countries, more computers per student were available in private schools (ratio = 0.97) than in public schools (ratio = 0.80) (OECD, 2020). Additionally, students who attend rural or regional schools face further technological challenges due to the lower levels of adoption which can be attributed to lack of infrastructure (Perrin, 2019).

### Expectations and perceived experience

The literature on consumer satisfaction advances several theories that could be applicable to our data. The Expectancy-Disconfirmation Paradigm (EDP) (Oliver, 1977, 1980) is one of the most promising theoretical frameworks to understand the relationship between expectations and perceived experience. It has been used in various industries, for example in tourism to investigate customer satisfaction (Wang & Davidson, 2008), in the field of public services to investigate citizen satisfaction (Lee et al., 2022; Zhang et al., 2021), in health services to investigate patient satisfaction in an emergency department (Cassidy-Smith et al., 2007). In an educational context, EDP has been used to study student engagement and expectations about discussion groups or online homework software (Schwarz & Zhu, 2015) and student satisfaction with a course (Appleton-Knapp & Krentler, 2006), amongst others. We find EDP particularly relevant to this paper. In essence, the EDP proposes that people form expectations about future performance, whether this is the purchasing of a product, or an experience, for example, learning experience as in our case. This expectation level becomes the standard against which the experience is judged. If the experience matches the expectations, then confirmation occurs. If there is a difference between expectations and experiences, then there is disconfirmation, which can be positive or negative. If the experience is better than what was expected, the positive disconfirmation results in satisfaction. If the experience is not as good as what was expected, the negative disconfirmation causes dissatisfaction.

Research findings confirm that expectations influence perceptions of the learning environment (Könings et al., 2008; Lizzio et al., 2002) and these perceptions affect satisfaction with the learning process and students’ performance (Struyven et al., 2008). Examination of past literature shows lack of recent studies focusing on student expectations of digital technologies in the higher education environment as informed by their school experience. Considering a drastic change in educational environments caused by the pandemic, change in students views on learning options, their expectations of digital technologies and their discovery of new technologies need to be studied to inform universities and their teaching staff on creating learning environments of the future.

### Expectations, perceptions and technology

Students are used to utilising different technologies for a variety of tasks (Pinto & Leite, 2020). The vast majority of students expect and have access to mainstream technology such as email, instant messaging, and social media for both private and study purposes (Bullen & Morgan, 2015; Junco & Cole-Avent, 2008). Based on the use of technology in schools and in private lives, students form their expectations of use of technology at universities (Jackson et al., 2011). Students do not use new technologies just because they become available, they choose technology based on how they recognise the value of technology on offer (Gosper et al., 2013). They use technology that provides convenience in performing tasks (Bansah & Agyei, 2022), having access to information (Hassan & Masoud, 2021; Lausa et al., 2021) and connecting them to staff and peers (Martin et al., 2020).

Previous studies on technology usage by university students discovered that most students use more than one device for their studies (Brooks & Pomerantz, 2017). They also showed how the use of technology evolved including the aspects providing insights into how students view technology and take advantage of what is on offer to improve their learning as well as academic achievements (Galanek et al., 2018). For many years, laptops and smartphones have been most used and perceived as extremely important in supporting the learning process (Crook et al., 2013; Dabbagh et al., 2019; Galanek et al., 2018; Henderson et al., 2015). Schools and universities heavily rely on learning management systems (LMS), however LMS have been teacher-centred and mainly used for distribution of information (Henderson et al., 2015; Janossy, 2008; Lacka & Wong, 2021). At the same time only a limited number of students have access and are experienced in using latest specialised technologies, such as Augmented Reality (AR) and Virtual Reality (VR), for their studies (Galanek et al., 2018).

## Methodology

In this study, we aim to determine whether there is a digital disconnect between expectations of first-year university students and their prior experiences in the final two years of high school. This study employed a mixed method research approach (Johnson & Onwuegbuzie, 2004). As only direct sampling from the university itself can provide insights into students’ views and expectations of the use of technology (Haywood et al., 2004), an online questionnaire was distributed to all on campus second-year students from a public university in Melbourne Australia.

### Data Collection Questionnaire

Questionnaires are commonly used for collecting data from a large number of participants in a short period of time. It is a common practice to analyse self-reported uses of technology for academic purposes through a questionnaire (Pike, 2011). The second-year students were contacted via email with the opportunity to participate in the online questionnaire which asked questions about their first-year experience of digital technology usage and their previous experience with digital technology at senior high school. The questionnaire posed 28 questions including questions about the demographics of the participant, self-reported ability to use technology, their expectations of technology use at university compared to actualities. A combination of close and open-ended questions were deployed to collect both quantitative and qualitative data from a large sample of students. The open-ended questions were analysed using thematic analysis (Guest et al., 2012). Thematic analysis “can be used to identify patterns within and across data in relation to participants’ lived experience, views and perspectives, and behaviour and practices” (Clarke & Braun, 2014, p. 297) which is in line with the aims of this research. The thematic analysis was implemented as described by (Braun & Clarke, 2006). Close-ended questions results were presented using statistics to provide an overview of student expectations and the relationship between demographics (i.e. gender, type of school and major field of study/faculty of study) and expectations.

### Participants

Table 1 shows the demographic profile of the participants. The total number of respondents to the survey was 422, however the survey was programmed in such a way that if a respondent did not meet the selection criteria they were disqualified and could not proceed. As a result, the number of completed responses significantly dropped to 189. The following criteria were applied for exclusion:

**Table 1:** Demographics of the participants

Profile	Category	Number *(n)*	Percentage (%)	University Wide – second year students’ enrolments
**Faculty**	Faculty of Science, Engineering & Technology (FSET)	60	31.75%	27.28%
	Faculty of Health, Arts & Design (FHAD)	96	50.79%	53.54%
	Faculty of Business & Law (FBL)	33	17.46%	19.18%
**Secondary School location**	City-based Metropolitan	125	66.49%	
	Regional	49	26.06%	
	Rural	14	7.45%	
**Type of secondary school attended**	Government	80	42.32%	
	Independent	51	26.98%	
	Catholic	58	30.69%	

*n = 189*

- not consenting to the questionnaire (six respondents did not consent)
- having undertaken less than six subjects of study physically on campus (36 respondents undertook less than six subjects).
- graduation from secondary school was outside 2017–2019 (95 respondents graduated before 2017, 0 respondents graduated in 2019 or later).
- aged under 18 or over 20 (1 respondent was under 18 and 88 respondents were older than 20, 20 of them also graduated before 2017).
- not fully completing the questionnaire (27 respondents abandoned the questionnaire closer to the middle).

The criteria were applied to ensure that the participants in the survey had the opportunity to be exposed to university teaching and that digital technologies were used in facilitating delivery. We also wanted to ensure that the students were recently exposed to technology in high school (since 2017) and that there was not a significant gap between high school and university in which technology would have change dramatically.

The total number of respondents to the questionnaire (*n = 189*) were from the three Faculties – ‘Science, Engineering and Technology’ (31.75%), ‘Health, Arts and Design’ (50.79%) and ‘Business and Law’ (17.46%) as summarised in Table 1. As can be seen in column 5 of Table 1, slightly more than half of the enrolled students (53.54%) were studying in the Faculty of Health, Arts and Design (FHAD) and this is also reflected by the corresponding number of respondents being from FHAD. The distribution of respondents across the three faculties is proportionally close to the distribution of enrolments among the three faculties. Among the respondents more females (60.85%) than males (35.97%) answered the questionnaire.

The students who participated in this questionnaire were enrolled in a variety of courses (see Table 2).

**Table 2:** University courses of participants

Courses	Number of Students	% of total respondents
Accounting	2	1.06%
Animation	6	3.17%
Architecture	2	1.06%
Arts	7	3.70%
Aviation	4	2.12%
Business	19	10.05%
Computer Science	14	7.41%
Criminology	4	2.12%
Design	23	12.17%
Education	1	0.53%
Engineering	29	15.34%
Film & Tv	16	8.47%
Health Science	17	8.99%
Information & Communication Technology	3	1.59%
Law	8	4.23%
Media Communication	11	5.82%
Nursing	4	2.12%
Psychology	11	5.82%
Science	4	2.12%
Screen production	3	1.59%
Sports Exercise	1	0.53%

*n = 189*

## Data Analysis

The main research question of this study was:

*Is there a digital technology disconnect between expectations and actual experiences when students get to first year university from high school?*

In addition, the research explored whether there were significant associations between:

- students’ expectations of technology usage and evaluation of whether these expectations were met;
- gender and students’ expectations on use of digital technology;
- the type of school our respondents were from (i.e. government, independent, catholic) with their expectations of technology and whether these expectations were met;
- the faculty where a student studies and evaluation of whether technology expectations were met.

Since two categorical variables needed comparison, Pearson Chi-square test of independence was used. Chi-square analysis has been used for exploratory data analyses in similar studies to examine associations in technology uses and demographic parameters of the students (Kennedy et al., 2010; Selwyn, 2008; Sharpe et al., 2019). Since the data had a high number of cells with expected count below 5, the Fisher-Freeman-Halton Exact Test (Agresti, 2003; Freeman & Halton, 1951) was used with statistical significance level defined at *p* < 0.05. All quantitative tests were conducted using SPSS.

Students’ comments were used to provide additional insights into statistical findings. All student comments were marked with the student profile including gender, assigned Faculty at university, the type of degree, whether their high school was metropolitan or regional and the type of school attended.

Students’ expectations of technology before the start of their university studies are summarised in Table 3. Most students (91.5%) expected more advanced or innovative digital technology used at university, while no one expected less than what they had experienced at high school. Out of the 173 students who indicated that they expected more technology and innovation at university, 43% attended Government schools, whereas students who attended Catholic schools (29%) and Independent schools (28%) had almost equivalent expectations. This almost equal distribution between Catholic and Independent Schools can perhaps be attributed to the level of school funding and resources at these types of schools.

**Table 3:** Students’ perceptions of their expectations before coming to university

Expectations	Number (*n)*	Percentage (%)	Catholic Students	Government Students	Independent Students
I expected it to be the same as secondary school	10	5.29%	6 (3.17%) (60%)	1 (0.53%) (10%)	3 (1.59%) (30%)
I expected it to be more advanced than secondary school	117	61.90%	33 (56.90%) (28.2%)	52 (65.82%) (44.4%)	32 (62.74%) (27.4%)
I expected it to be more innovative than secondary school	56	29.63%	17 (29.31%) (30.4%)	23 (29.11%) (41.2%)	16 (31.37%) (27.4%)
I expected it to be less advanced than secondary school	0	0 (0%)	0 (0%)	0 (0%)	0 (0%)
I expected it be less innovative than secondary school	0	0 (0%)	0 (0%)	0 (0%)	0 (0%)
I had no expectations	5	2.65%	2 (3.45%)	3 (3.80%)	0 (0%)
I don’t remember	1	0.53%	0 (0%)	1 (1.27%)	0 (0%)
Total	189	100%	58	80	51

Having completed first year of studies at university, students were asked to comment on whether their technology expectations were met (see Table 4). 28.6% of students confirmed that their expectations had been exceeded. The breakdown was identical for students who attended Catholic and Government schools with 10.58% and much lower for Independent schools (7.4%). 55% of students indicated that their first-year experience had matched their expectations with the following breakdown: 23.28% Government Schools, 16.93% Catholic Schools, and 14.81% Independent Schools.

**Table 4:** Students’ expectations of technology after they completed Year 1 of university

Expectations	Number (*n)*	Percentage (%)	Catholic Students	Government	Independent
Has exceeded my expectations from secondary school	54	28.57%	20 (10.58%)	20 (10.58%)	14 (7.40%)
Has matched my expectations from secondary school	104	55.03%	32 (16.93%)	44 (23.28%)	28 (14.81%)
Is poorer than what I expected in secondary school	10	5.29%	3 (1.59%)	4 (2.17%)	3(1.59%)
Is different to what I expected at secondary school, but neither poorer or better	21	11.11%	3 (1.59%)	12 (6.35%)	6 (3.17%)
Total	189	100%	58 (30.69%)	80 (42.33%)	51 (26.98%)

Some previous studies examined the association between gender and students’ expectations and actual use of digital technology (Henderson et al., 2015; Meelissen & Drent, 2008; Sharpe et al., 2019; Smith et al., 2009). In the present study, the majority of respondents were female (61%), 36% were male and very small number (3%) identified as non-binary or preferred not to answer. Using a Fisher-Freeman-Halton Exact test for data analysis showed no significant association between gender and use of technology expectations (*FFHET (N = 189) = 27.566, p = 0.147*). Similarly, there is no significant association between gender and evaluation on how the expectations have been met (*FFHET (N = 189) = 12.266, p = 0.425*). These results are in line with the previous findings (see for example Jones & Ramanau 2009). Data analysis using a Fisher-Freeman-Halton Exact test showed no significant association between students’ expectations and evaluation of whether these expectations were met (*FFHET (12, N = 189) = 17.353, p = 0.090*).

As part of demographics analysis we examined the relationship between the type of school our respondents attended (i.e. Government, Independent, Catholic) with their expectations of technology usage and whether those expectations were met. As summarised in Table, 1 42.32% of respondents attended Government schools, 26.98% - Independent schools, 30.69% - Catholic schools.

Applying Fisher-Freeman-Halton Exact test the collected data shows no significant association between the school type and expectations on utilising technology for studies (*FFHET (N =189) = 17.780, p = 0.101*), nor between the school type and evaluation of whether these expectations were met (*FFHET (N =189) = 6.727, p = 0.644*).

Students’ comments provided insights into statistical findings on high expectations of utilising technologies at university level regardless of the type of school they attended. They had expectations that university would be well resourced with technology compared to their time in high school and be heavily reliant on technology therefore classes would be impersonal as can be seen by the selection of comments.

*I expected my university to have more funding than my high school did, and therefore better resources. I also expected university to be a lot less personal (due to one person teaching a far greater number of students) and therefore online resources to be utilised a lot more due to ease of accessing a greater number of students at once.* – **[Female, FBL, Law, Attended Government school- Metropolitan, Comfortable using computers]**

*I thought that I’d be using, and installing, varying programs onto my computer for different classes. I believed, the resources I’d be using would be more advanced than those of basic secondary school, and I’d be using new software and or programs.* – **[Female, FHAD, Health Science, Attended Catholic School - Metropolitan, Very comfortable using computers**]

*My expectations were that universities were focused on future job markets and preparedness for such industries. As such, I expected not just current technologies, but emerging ones to be utilized during the course.* – **[Male, FHAD, Design, Attended an Independent School- Metropolitan, Proficient is using computers]**

In the following subsections we describe the themes identified through thematic analysis.

### Funding

Students voiced several assumptions, particularly around funding and technology expectations. The views held by students indicated that because universities were larger, they had more funding and as a result students had higher expectations of sophisticated technology access and use than what they were used to at high school. This was affirmed by their comments that they thought the technology at the university would be “more advanced” or “more innovative” than their experience at high school. It was difficult to build a profile of the student that held these views as closer analysis showed they were studying in a variety of courses located in different faculties and they attended different types of high schools as can be seen below:

*University probably has more capital than my dingy public high school*. – **[Female, FSET, Computer Science, Attended a Government School - Metropolitan, Very comfortable with Computers]**.

*better funding at uni than my high school, also to learn how to use industry standard softwares.* – **[Female, FHAD, Film and TV, Attended Government School - Metropolitan, Proficient in using computers]**.

*University seems to be much more well-funded, so I expect that tech used would be more advanced than secondary.* – **[Male FSET Comp Science, Attended Independent School - Metropolitan, Proficient at using computers]**.

*Because universities have a higher budget.* – **[Male, FHAD, Design, Attended Catholic school - Metropolitan, Proficient in using computers]**.

### Learning at University

Students already had preconceived ideas of technology use at university. They believed that it would be more advanced than their time in high school. They also had expectations that university would be well resourced with technology compared to their time in high school, and they expected that there would be heavy reliance on technology and therefore classes would be impersonal as can be seen by the selection of comments:

*Students increase their digital literacy skills as they grow older; hence I expect university classes to utilise more advanced technology that high schoolers would not understand.* – **[Male, FSET, Computer Science, Attended Catholic School - Regional, Proficient in using computers]**.

*At high school there was more writing by hand, I expected a university of technology to be quite advanced.* – **[Male, FSET, Engineering, Attended Catholic, Metropolitan, Comfortable in using computers]**.

*I expected university to be more online based and self-taught than secondary school, therefore believed the technologies would be more advanced.* – **[Female, FHAD, Psychology Attended a Government school - Metropolitan, Comfortable in using computers]**.

### Specific digital technologies

To reiterate, the respondents’ distribution across the university’s three Faculties was as follows. The majority of students were from the Faculty of Health, Arts & Design (FHAD) – close to 51%, 32% were from the Faculty of Science, Engineering & Technology (FSET) and 17% from the Faculty of Business & Law (FBL) (see Table 1). Past studies examined discipline-based differences in technology use patterns. Sharpe et al., (2019) identified students in Information Technology degrees as intensive users of technology which is to be expected. A study by Selwyn (2008) that focused on the Internet as technology, identified quite a few degrees such as Business, Law, Social Sciences where students heavily used technology. Using a Fisher-Freeman-Halton Exact test for data analysis showed no significant association between the Faculty where a student studied and evaluation of whether technology expectations were met (*FFHET (N = 189) = 7.967, p = 0.225*). Therefore, our findings indicate that modern generations of students use technology regardless of the degree they study. The differences in findings could be explained by the years passed since Selwyn’s study and the narrow focus of that previous study on the Internet as technology.

Students expectations of specific technologies for their studies was analysed in relation to the faculty where they study (Table 5). The number of students that expected to study in computer labs was proportionally greater in FSET compared to FHAD (*p = 0.000*), which in turn was proportionally greater than in FBL (*p = 0.006*). Proportionally larger number of FSET students than FHAD students reported that they expected the use of online quizzes (e.g. Kahoot) in their studies (*p = 0.017*). Understandably, the proportion of students that expected to use programming/coding technologies was greater at FSET than at FHAD (*p = 0.000*) or at FBL (*p = 0.001*). Similarly, the proportion of students that expected to use robotics/Arduinos technologies was greater at FSET than at FHAD (*p = 0.000*) or at FBL (*p = 0.006*).

**Table 5:** Students’ expectations of technology by Faculty affiliation

			Which Faculty are you associated with?			
			Faculty of Science, Engineering and Technology (A)	Faculty of Health, Arts and Design (B)	Faculty of Business and Law (C)	Total
**Tech expectations by Faculty** In this question, we ask that you think back to your last 3 years of secondary school (high school). Please select: a) Column 1: all the technologies that you used as a student in secondary school b) Column 2: all the technologies that you thought at the time you would use at as a university student Please select as many responses as is appropriate for each column by clicking on all the relevant buttons.	- Online quizzes (i.e. KAHOOT) Technologies you EXPECTED to use at University as a student	Count	38 B( .017)	39	20	97
	- Programming/Coding Technologies you EXPECTED to use at University as a student	Count	44 B( .000) C( .001)	27	12	83
	- Robotics/Arduinos Technologies you EXPECTED to use at University as a student	Count	18 B( .000) C( .006)	6	1	25
	Classes with computer labs Technologies you EXPECTED to use at University as a student	Count	55 C( .000)	74 C( .006)	16	145
	Total	Count	60	96	33	189

Results are based on two-sided tests. For each significant pair, the key of the category with the smaller column proportion appears in the category with the larger column proportion

a. Tests are adjusted for all pairwise comparisons within a row of each innermost subtable using the Bonferroni correction

### Student Expectations

Students were asked whether their technology expectation as a student at university has been met. Most of the respondents (91.53%) expected more advanced or different technology, however none expected less as can be seen in Table 4. Most (83.6%) stated that their expectations were matched or exceeded, while of the 11.11% who said it was different (*n = 21*), only 2 were negative, 2 were mixed with both a positive and negative comments, 4 were positive about the difference, and the rest were neutral or had no comment. The results indicate that the respondents to this questionnaire did not encounter a disconnect in use of digital technologies between high school and university. Where the expectations differed from what they used at school, the response was mostly positive and favourable. Comments such as:

*There were technologies that I was unaware existed that I have seen/used during my course that has allowed me to better understand what I was being taught.* – [**Male, FSET, Science, Attended a Catholic School- Rural, Proficient in using computers]**.

Another student commented that:

*Swinburne has so many amazing facilities related to technology. I remember being taken on a tour in first year, and was blown away by what they had available to students.* – **[Female, FHAD, Design (Communication Design), Attended an Independent School - Rural, Comfortable with computers]**

To counter balance the positive comments, the two negative comments from students are included below:

*The technology available has not ‘wowed’ me as something I did not expect in the business faculty*. - **[Female, FBL, Business, Attended a Catholic School- Metro, Comfortable with computers]**.

*Everything has been horrible with the new online learning. Online classes are horrible and difficult to manage*. - **[Female, FHAD, Health Science, Attended a Government School- Metro, Ok with computers]**.

We can conclude that those who wanted to use some type of digital technology actually used it. Those who started using a specific type of technology at school continued using it at university.

## Discussion

In the climate of a worldwide pandemic where the Australian Higher Education sector suffered severe financial constraints, we wanted to find out if students perceived a digital technology disconnection between their expectations at high school and their experiences in first year of university. Our initial assumption was that the financial cuts might have had an impact on how students perceived the use of technology in their university education, and that this perception might be influenced by their previous experiences in high school. We expected to find a disconnection in students’ expectations and experiences and we assumed that this gap might vary significantly depending on the type of school they attended, their gender or other relevant variables. Hence, we aimed to answer the question: *Is there a digital technology disconnect between expectations and actual experiences when students get to first year university from high school?*

Based on our sample, the vast majority of students (91.53%) expected digital technologies at university to be more advanced or innovative than their school experience, which is in agreement with the literature that points to students’ attitudes towards digital technologies as important tools that can enrich their study experiences (Henderson et al., 2017; Novikova et al., 2021). Students in our sample gave several reasons why they had these expectations:

(1) Universities are better funded than schools and therefore it is expected that the technologies will be more advanced.

(2) The work done at universities requires more advanced technologies (e.g., industry standard software).

(3) Learning at university is expected to be more impersonal and reliant on technological resources (e.g., online learning) for self-teaching.

(4) Learning at university is expected to be more advanced or specialised, which requires technologies that are usually not necessary at secondary school (e.g., preparing students for the job market).

(5) The university where the data was collected was a “university of technology”, hence the expectation was that the technology would be a pronounced feature.

Nevertheless, it was surprising to see that 94.71% of the students in our sample thought that their expectations were either matched (55.03% matched + 11.11% nor poorer nor better) or exceeded (28.57%). We would have expected that perhaps students coming from Independent schools would be more likely to experience a disconnection given that they would be used to more technology and their expectations would be greater and harder to satisfy. Our results showed that we cannot say that the type of school a student comes from, or the gender of the student, made a significant difference in the (dis)connection between expectations and actual experiences of the use of digital technologies.

Our students gave 3 main reasons to explain why they felt their expectations were met or exceeded:

(1) Some technological products at university were unknown to the student and/or the facilities related to technology were deemed very good.

(2) Access to technology was very easy (e.g., being able to borrow a laptop or using software without the need to buy a license).

(3) Good and easy to access technical support.

Hence, despite the financial difficulties and how these translated into restricted provision of technologies in education, most students still considered their experiences to be positive. They recognised the value in how new technologies that they did not know could be used in their respective fields of study (cf. Gosper et al., 2013) and how previously existing support was still adequate. A possible explanation for this overwhelming positive response from students might be the teachers’ “capacity to do great things for and with students […] despite sparse resources” (Jones & Kessler, 2020, p.1). Indeed, researchers have reported that during the pandemic many students realised the advantages of online learning and of being able to learn new technological tools, such as communication software and Cloud-based tools (Almahasees et al., 2021; Almusharraf & Khahro, 2020).

Of the 10 students that thought their experience was poorer than what they expected, most of them alluded to the human interaction and integration with technology. This suggests that there are some issues of curriculum development (e.g., use of software to bring meaning to complex concepts) and teacher professional development (e.g., development of digital literacies) that need to be addressed in specific areas (DeCoito & Estaiteyeh, 2022).

The results of this study are consistent with the Expectancy-Disconfirmation Paradigm (EDP). We saw many students *confirming* (i.e., expectations and experience matched) their expectations. Our results showed that those students who started using certain technologies in high school were able to continue using them at university. We can conclude that for these students, there was no disconnection in their transition to university in terms of the use of technologies.

There was also *disconfirmation*, both positive (28.57%) and negative (5.29%). Those whose expectations were exceeded talked about *satisfaction* at discovering new technologies or finding technical support very easy to access. Those whose expectations were not met showed *dissatisfaction* with the way their courses lacked the level of technology they anticipated or how the teaching focused more on the theory rather than on the technological elements. The latter students were studying courses where technology is expected to play an important role (e.g., Computer Science, Data Analytics) and therefore we can assume that the difference between their expectations and their experiences of technology was large. According to the EDP, a positive disconfirmation would cause the experience to be highly appraised whilst a negative disconfirmation would result in a poor evaluation of the experience. Therefore it is imperative for universities to continue to pursue and implement new technologies and also provide professional development for their staff.

## Conclusion

Modern day tertiary institutions provide mainstream digital technologies for staff and students. These technologies support teaching delivery and students’ learning. Institutions need to make good decisions on which technologies are core for creating engaging learning environments. These decisions require regular reviews and these reviews need to rely on sound information which should also reflect students views, perceptions and expectations of digital technologies for successful learning.

Our findings demonstrate that most students perceived little disconnect between the digital technology they used in high school and that at tertiary level. The majority of students seemed satisfied and even positively surprised by the use of digital technology in their university courses. Those students who wished to use some type of digital technology actually used it, whereas those who started using a specific type of technology at school continued using it at tertiary level. Most students expected more advanced or innovative digital technology used at university, while no-one expected less than what they had experienced at high school. Most students said their expectations of digital technologies were matched or exceeded at university when compared to their school experience.

Although students thought that the technology at tertiary level would be “more advanced” or “more innovative” than at high school, it was difficult to build a profile of the student that held these views. Regardless of the demographics parameters, such as gender, school type and field of study, students expected to use a variety of digital technologies, including the technologies they used for their studies and in private lives and they also expected to use new advanced technologies. Majority of students indicated that their expectations have been met.

This study has some limitations. 189 responses to the questionnaire is acknowledged to be on the lower side, however, if there is no significant differences in respondents and non-respondents demographics and the demographics parameters are not significantly skewed, low response rate should not be a cause for concern (Sivo et al., 2006). Furthermore, we collected our data at the beginning of the Covid-19 pandemic which we assume contributed negatively to the response rate. We also acknowledge that we do not know if the pandemic influenced some of the student responses by highlighting the (un)preparedness of the higher education sector to move to online-based teaching. Also the uneven distribution of respondents between faculties with half of the respondents coming from FSET could potentially skew the results towards higher expectations of technology use by students in technical courses.

As more studies on the impact of the Covid-19 pandemic begin to be published, a clearer picture might emerge of how student expectations changed as a result. Their satisfaction with current technologies may decrease if the institutions do not implement newer digital technologies. Tertiary institutions cannot become complacent with their current digital technologies, but must continue to invest, in both the newest digital technologies together with the professional development of their staff in their use and application. However, these decisions on digital technologies need to be grounded in data, especially data on students expectations and engagement which is in line with the previous research findings (Kuh, 2003).

## Data Availability

The datasets generated during and/or analysed during the current study are not publicly available due to not being part of ethics approval but are available from the corresponding author on reasonable request.
